# Supplementary material for: Extreme haplotype variation in the desiccation-tolerant clubmoss Selaginella lepidophylla
Source: Nat Commun. 2018 Jan 2;9:13. doi: 10.1038/s41467-017-02546-5 (PMC5750206; doi:10.1038/s41467-017-02546-5)
Supplement: Supplementary file 1 — Supplementary Information [file 41467_2017_2546_MOESM1_ESM.pdf]

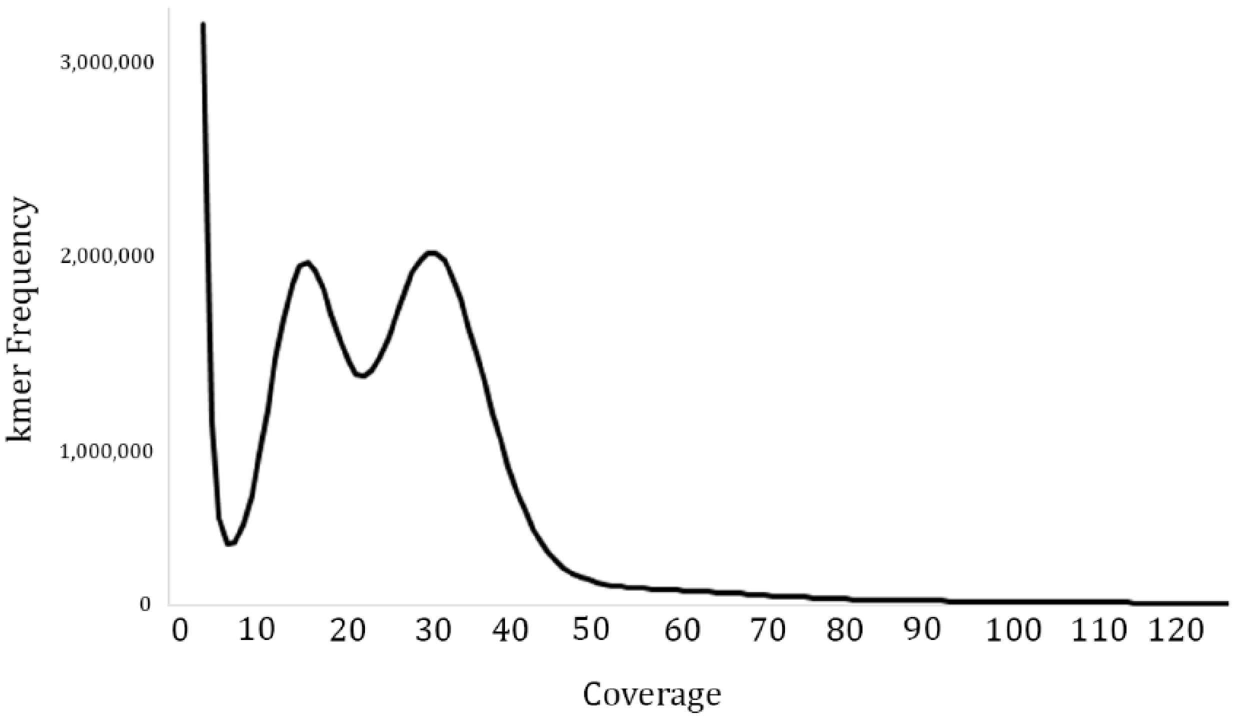

Supplementary Figure 1. High within genome heterozygosity of *S. lepidophyla*. A histogram of kmer frequency (k= 27) of Illumina reads is plotted. Frequency patterns display a bimodal curve indicating a high rate of within genome heterozygosity with an estimated diploid genome size of 109 Mb.

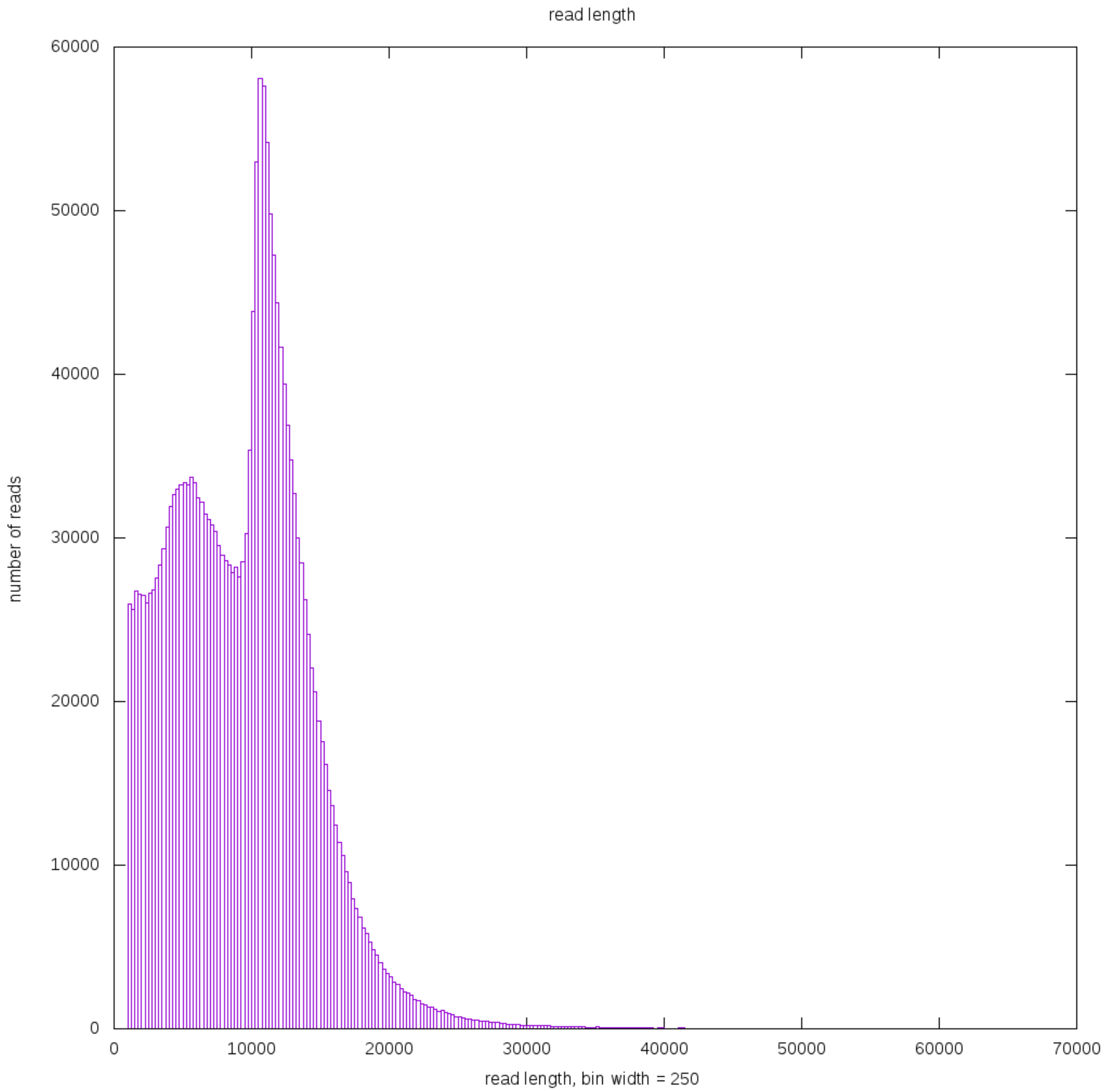

Supplementary Figure 2. Histogram of Subread length distribution of raw PacBio reads. The read length (bp) of raw PacBio reads is plotted on the x-axis.

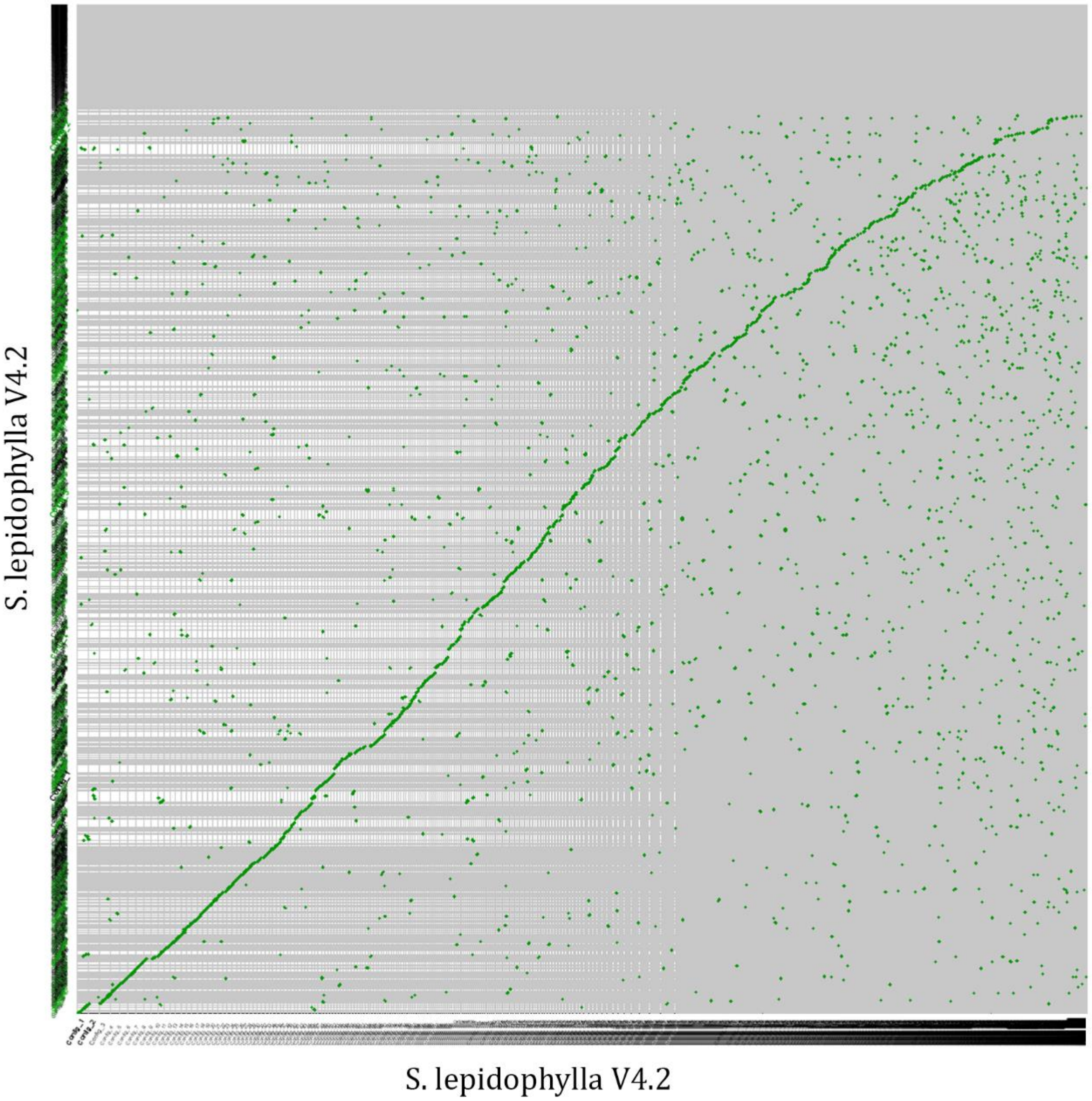

Supplementary Figure 3. Macrosytenic dot plot showing haplotype specific assembly across the S. lepidophylla genome. Green dots represents syntenic regions between the two haplotypes.

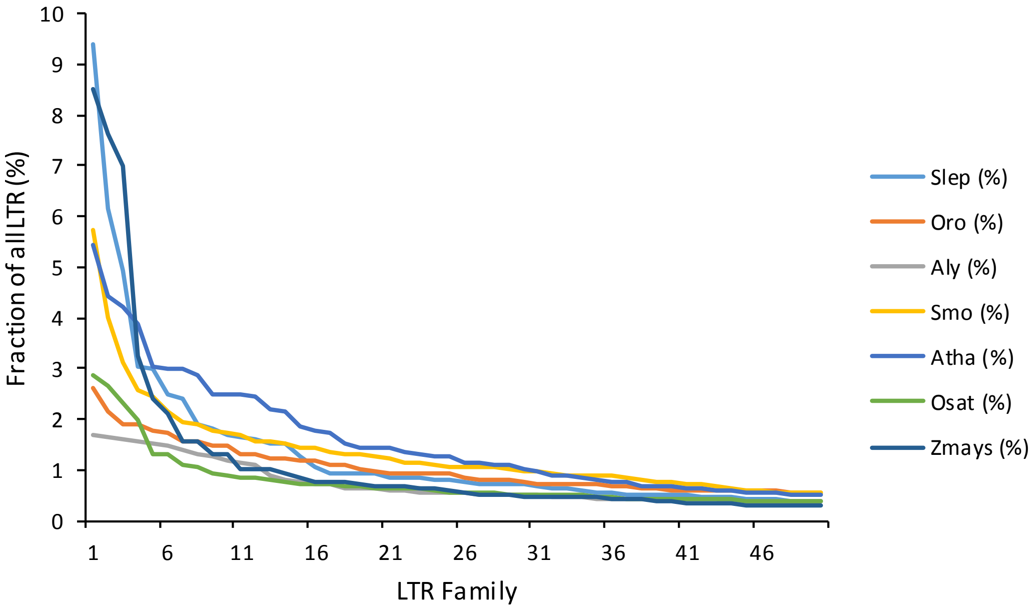

Supplementary Figure 4. Replicability of LTR families in seven plant genomes. Replicability of an LTR family is defined by the proportion of the size of an LTR family to all LTR sequences in the genome. The top 50 replicability of LTR families is plotted for each genome. *S. lepidophylla* has the most replicable LTR families among the seven genomes, with a single LTR family contribute to 9.3% of all LTR sequences. Slep, *S. lepidophylla*; Smo, *S. moellendorffii*; Oro, *Oropetium thomaeum*; Atha, *Arabidopsis thaliana*; Aly, *A. lyrata*; Osat, *Oryza sativa*; Zmays, *Zea mays*.

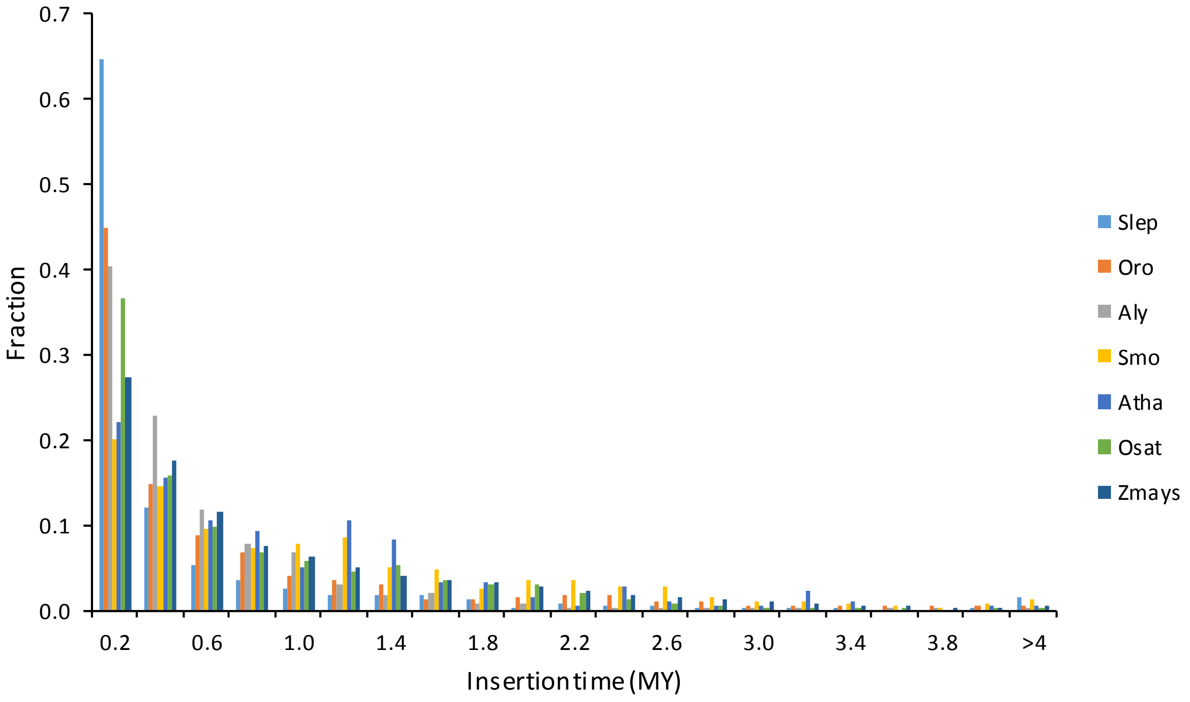

Supplementary Figure 5. Insertion time distribution of seven plant genomes. While most of intact LTR retrotransposons were inserted within 4 million years (MY) ($u=1.3 \times 10^{-8}$ per bp per year, sequence divergence is corrected using the Jukes-Cantor model for non-coding sequences), the *S. lepidophylla* genome has many more young LTR retrotransposons, with ~25% more LTRs inserted within 0.2 MY comparing to other genomes. Y-axis denotes the proportion of the respective category in all cases. Slep, *S. lepidophylla*; Smo, *S. moellendorffii*; Oro, *Oropetium thomaeum*; Atha, *Arabidopsis thaliana*; Aly, *A. lyrata*; Osat, *Oryza sativa*; Zmays, *Zea mays*.

a

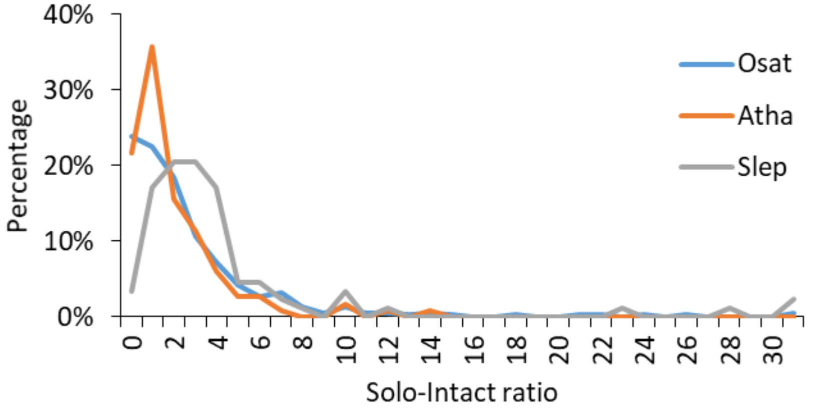

b

	LTR length*	Age (MY)	S/I
S. lepidophylla	512^{a}	0.371^{a}	4.06^{a}
O. sativa	535^{a}	0.924^{b}	2.71^{b}
A. thaliana	445^{a}	0.938^{b}	1.80^{c}

Supplementary Figure 6. Comparison of solo-intact (S/I) ratio of LTR retrotransposons in three plant genomes. (a) Distribution of S/I ratio of each LTR-RT family for three plant genomes. (b) Means of LTR length, LTR age, and S/I ratio of each genome using the length-filtered dataset. Different letters in each column indicate statistical significance by pairwise *t*-test ($P < 0.05$). Osat, *Oryza. sativa*; Atha, *Arabidopsis thaliana*; Slep, *S. lepidophylla*.

*To remedy the LTR length effect in intra-element recombination, the 95 percentile of LTR length in *Selaginella lepidophylla* (90-1540 bp) was used to screen for LTR-RT families that fell within this size range in the rice and Arabidopsis genomes.

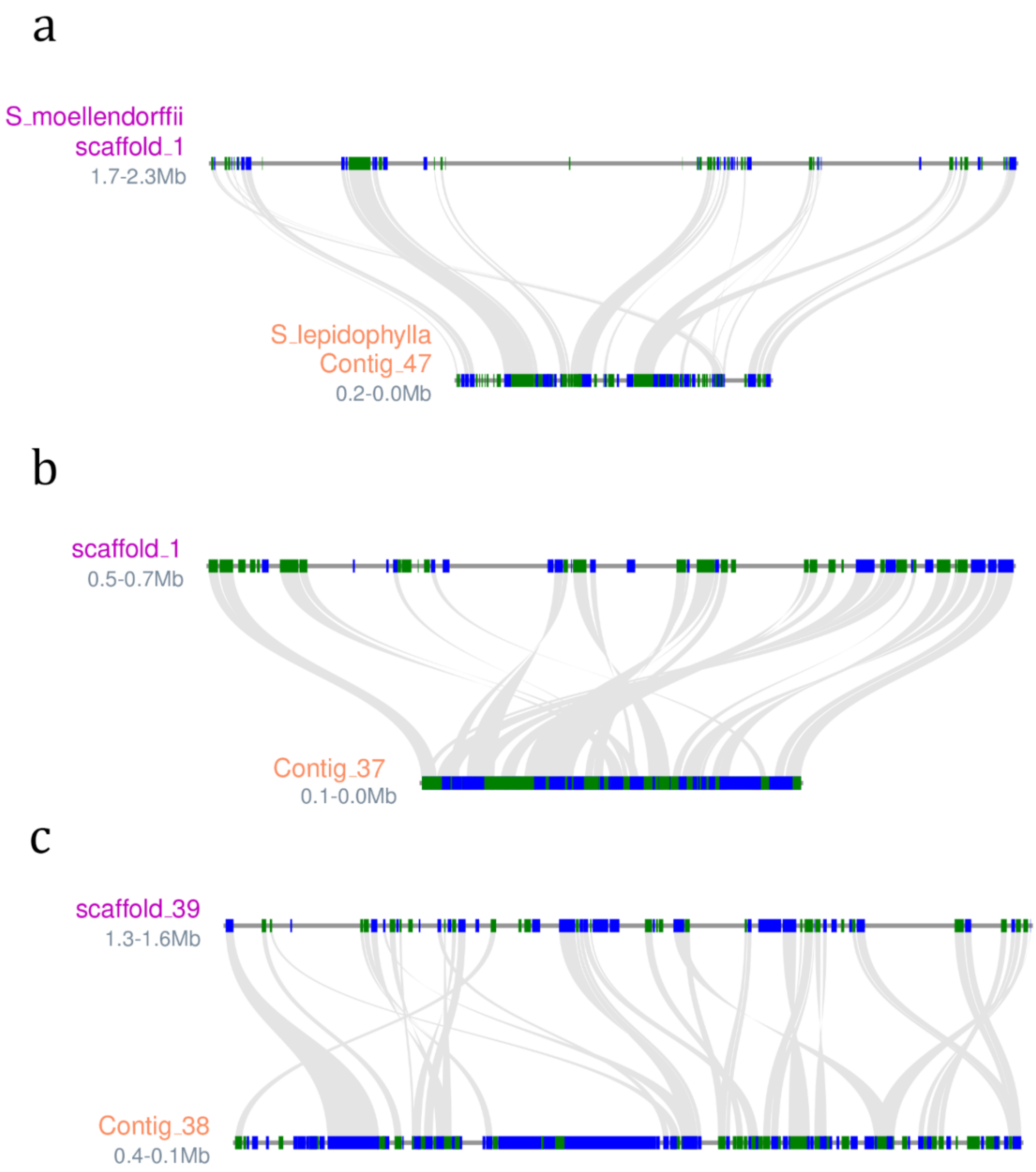

Supplementary Figure 7. Microsynteny between the *S. lepidopylla* and *S. moellendorffii* genomes. Genes are colored by orientation with blue correlating to forward and green reverse. Syntenic gene pairs are connected by grey lines.

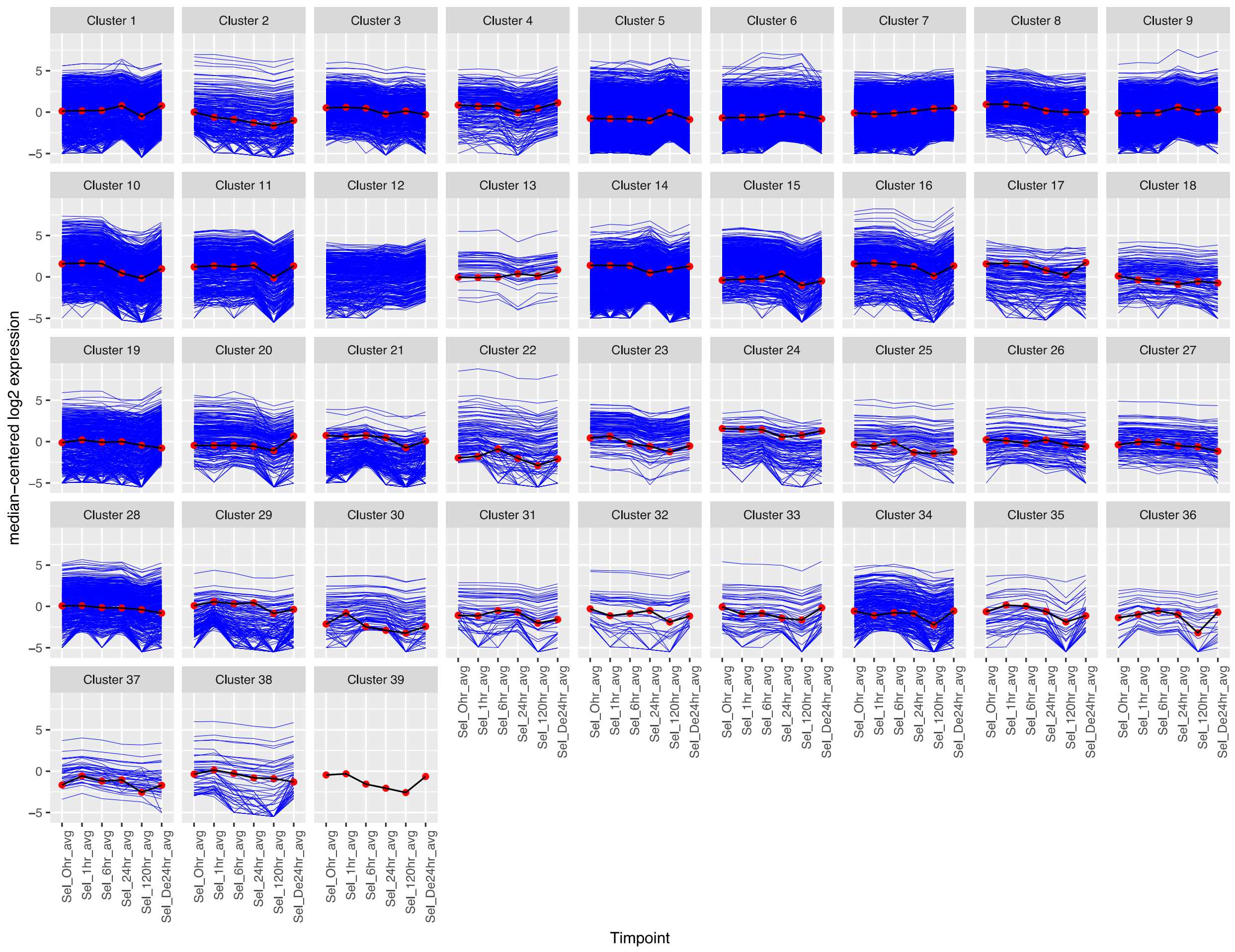

Supplementary Figure 8. WGCNA-based co-expression clusters across the *S. lepidophylla* rehydration/desiccation timecourse. Median-centered Log2 TPMs are plotted for the complete set of 38clusters.

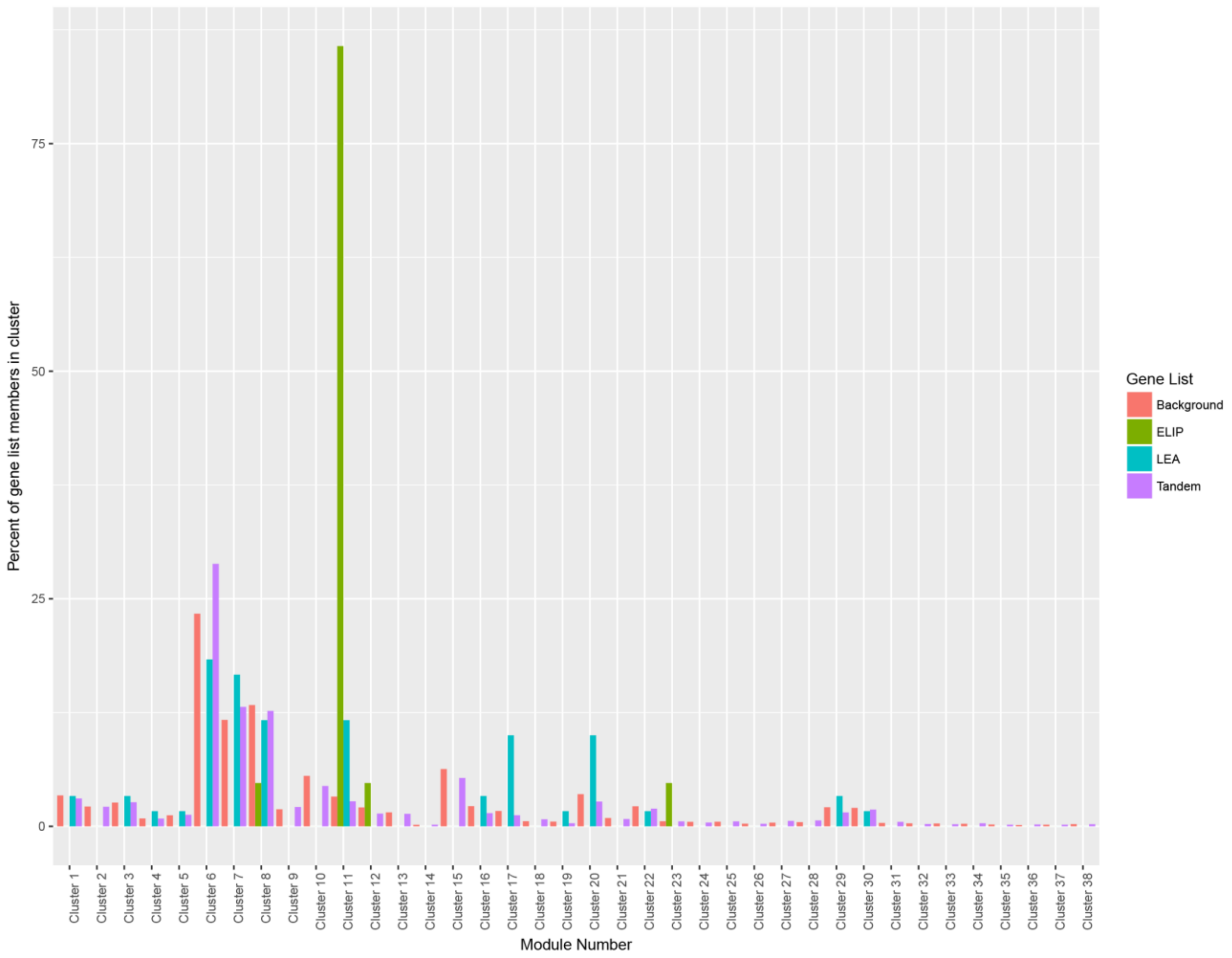

Supplementary Figure 9. Enrichment of gene co-expression cluster in tandem duplicates and desiccation related genes. The background, tandem gene, LEA, and ELIP frequencies are presented for each of the 38 coexpression modules.

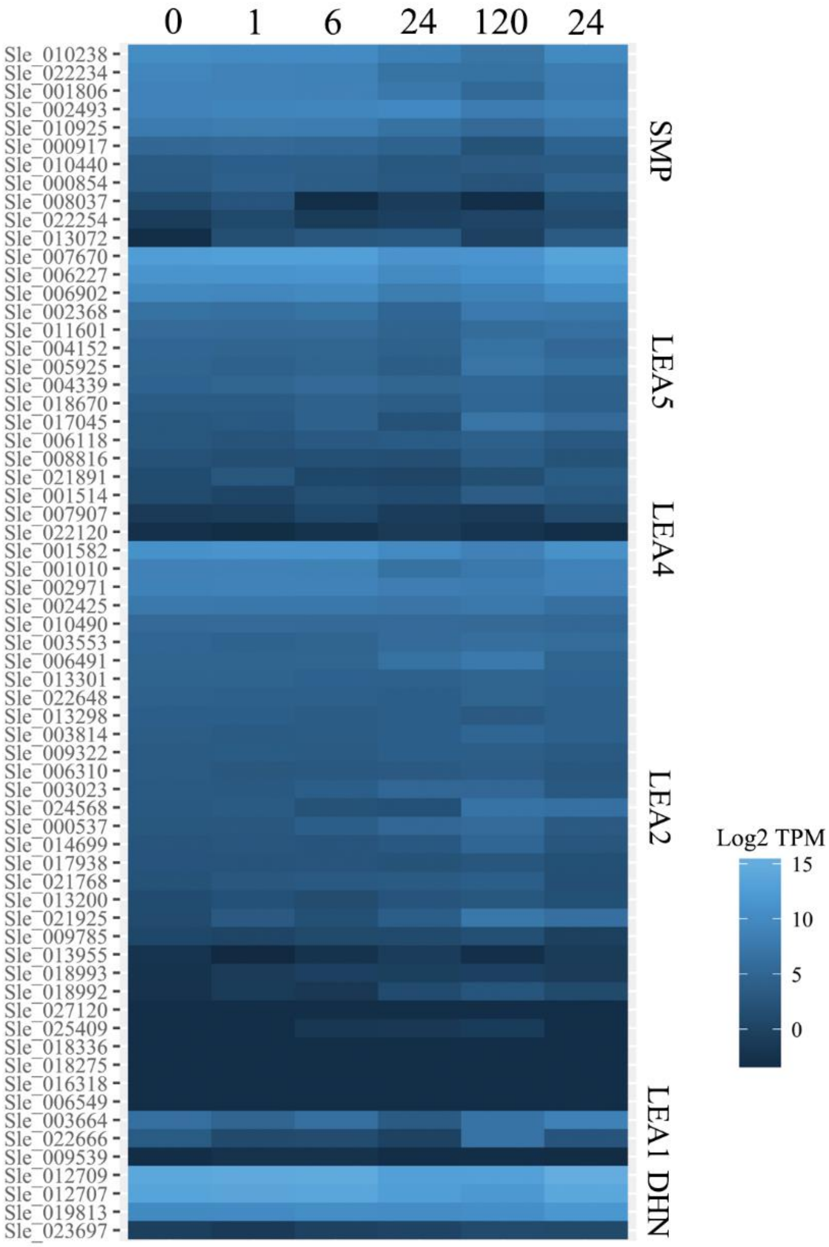

Supplementary Figure 10. Expression patterns of LEA proteins during the desiccation and rehydration process in *S. lepidophylla*. Log2 TPM (transcripts per million) is plotted for all 86 LEA proteins under the 6 time-points. LEA, Late Embryogenesis Abundant; SMP, Seed Maturation Protein; DHN, Dehydrin.

Supplementary Table 1. Summary statistics of the Illumina based *S. lepidophylla* genome assembly.

Assembly metric	#
Number of sequences	21,282
Total size of sequences	53,779,066
Longest sequence	42,938
Shortest sequence	1,000
Number of sequences > 10	0 (0%)
Number of sequences > 100	0 (0%)
Number of sequences > 1K	21,039 (98.86%)
Number of sequences > 10K	243 (1.14%)
Number of sequences > 100K	0 (0%)
Number of sequences > 1M	0 (0%)
Number of sequences > 10M	0 (0%)
Mean sequence length	2,526
Median sequence length	1,819
Mode of sequence lengths	1,002
N50 sequence length	3,022
L50 sequence number	5,143
N90 sequence length	1,253
L90 sequence number	16,454

Supplementary Table 2. Final PacBio based *S. lepidophylla* genome assembly metrics.

Assembly metric	#
Number of sequences	1,149
Total size of sequences	122,531,738
Longest sequence	1430,794
Shortest sequence	2,325
N50 sequence length	163,247
N90 sequence length	42,531

Supplementary Table 3. Assessment of annotation completeness using BUSCO.

	S. lepidophylla	*S. moellendorffii*
Complete BUSCOs	292	290
Complete and single-copy BUSCOs	238	250
Complete and duplicated BUSCOs	54	40
Fragmented BUSCOs	8	8
Missing BUSCOs	3	5
Total BUSCO groups searched	303	303
**Total % complete**	**96.3%**	**95.7%**

Supplementary Table 4. Summary of transposable elements (TEs) and other repetitive sequences in the *S. lepidophylla* genome

	Super-family	No. of TE[a]	Coverage (Mb)	Fraction of genome (%)
Class I	LTR/*Copia*	1966	0.71	0.58
	LTR/*Gypsy*	18,959	11.66	9.52
	LTR/Unknown	6,008	3.64	2.97
	LINE	4,702	4.35	3.55
	**Total class I**	31,635	20.36	16.62
Class II	*En*/*Spm*/CACTA	242	0.07	0.06
	hAT	1,324	0.52	0.42
	Helitron	527	0.45	0.37
	MULE/*MuDR*/*Mutator*	2,633	0.85	0.70
	PIF/*Harbinger*/*Tourist*	186	0.08	0.07
	Sola	4,293	5.34	4.36
	Other	5,120	2.46	2.01
	**Total class II**	14,325	9.77	7.99
**Total TEs**		45,960	30.13	24.61
Tandem repeats		22,989	2.89	2.35
Other unknown repeats		19,900	8.35	6.82
**Total repeats**		88,849	41.37	33.78

[a]Intact and fragmented.

Supplementary Table 5. Summary of intact LRT retrotransposons in *Selaginella*.

	S. lepidophylla v4.2				***S. moellendorffii V1***			
	**Intact LTR #**	**Contribution**	**Genome content**	**Contribution**	**Intact LTR #**	**Contribution**	**Genome content**	**Contribution**
all	744	100%	13.07.%	100%	941	100%	30.70%	100%
Copia	17	2.28%	0.58%	4.43%	58	6.20%	0.80%	2.60%
Gypsy	572	76.88%	9.52%	72.84%	592	62.90%	17.20%	56.10%
Unknown	155	20.83%	2.97%	22.72%	291	30.90%	13.70%	44.60%

Supplementary Table 6. Detailed descriptions of regions with haplotype specific structural variation.

Haplotype 1	Haplotype 2	Start H1	End H1	Start H2	End H2	Haplotype SV	Start SV	End SV	Size (bp)	Orientation
Contig_1	Contig_45	570967	935971	25345	397879	Contig_45	876198	897063	20865	-
Contig_1	Contig_58	9188	384824	1	326719	Contig_1	73848	90490	16642	-
Contig_3	Contig_162	658740	814537	5254	180589	Contig_162	41141	50135	8994	-
Contig_3	Contig_162	658740	814537	5254	180589	Contig_162	54565	66105	11540	-
Contig_3	Contig_172	284598	340520	12373	87495	Contig_172	58549	84752	26203	+
Contig_3	Contig_311	144436	279562	1	111894	Contig_311	22204	31259	9055	-
Contig_3	Contig_418	842263	975475	1	87210	Contig_3	882019	933446	51427	+
Contig_4	Contig_499	446557	568327	1	70665	Contig_4	506670	545417	38747	
Contig_4	Contig_97	152663	371645	5721	249333	Contig_97	64303	118882	54579	+
Contig_4	Contig_97	152663	371645	5721	249333	Contig_97	154396	182993	28597	
Contig_4	Contig_212	609377	734421	1	143257	Contig_212	85548	108214	22666	-
Contig_4	Contig_438	768607	869024	1	83725	Contig_4	784020	813294	29274	-
Contig_5	Contig_103	774842	949566	43355	243028	Contig_103	84750	101742	16992	-
Contig_5	Contig_409	335290	392597	1	77123	Contig_409	16670	27393	10723	-
Contig_5	Contig_453	566615	633086	1	79520	Contig_453	18835	46288	27453	+
Contig_6	Contig_52	525828	878440	1	367130	Contig_52	172890	253269	80379	+
Contig_6	Contig_229	133298	300684	1	135847	Contig_229	197491	229020	31529	+
Contig_6	Contig_477	79775	139953	1	75872	Contig_477	24544	35018	10474	-
Contig_7	Contig_39	364953	775373	3	431357	Contig_7	691318	748059	56741	-
Contig_7	Contig_124	1	243664	11688	213890	Contig_124	159581	173566	13985	+
Contig_9	Contig_263	233182	372316	1	127773	Contig_263	303539	317336	13797	-
Contig_10	Contig_110	16982	206203	1	139926	Contig_10	106327	118861	12534	-
Contig_10	Contig_510	601391	671852	1	49878	Contig_10	633386	647134	13748	+
Contig_11	Contig_127	1	243081	1	204868	Contig_127	34334	51459	17125	-
Contig_11	Contig_247	577386	705787	1	132377	Contig_11	630397	652352	21955	-
Contig_11	Contig_444	273538	348985	2	78829	Contig_444	31377	42170	10793	-
Contig_12	Contig_69	26473	321544	1	310061	Contig_12	286076	304454	18378	+
Contig_12	Contig_204	456924	578013	1	147538	Contig_204	62549	71058	8509	+

Contig_12	Contig_204	456924	578013	1	147538	Contig_204	126320	137965	11645	+
Contig_13	Contig_179	309155	496838	74	161583	Contig_179	337493	393559	56066	+
Contig_13	Contig_179	309155	496838	74	161583	Contig_179	28512	66189	37677	+
Contig_13	Contig_202	3719	193856	1	148977	Contig_13	124510	143499	18989	+
Contig_13	Contig_249	183097	277128	230	132225	Contig_249	35011	86345	51334	+
Contig_14	Contig_177	120	161516	1	155510	Contig_14	101004	107467	6463	-
Contig_15	Contig_91	427651	653180	10169	259467	Contig_91	148794	191689	42895	-
Contig_15	Contig_252	221785	428627	1	131772	Contig_15	283838	348556	64718	-
Contig_15	Contig_382	1810	89279	3849	95472	Contig_382	52946	72739	19793	+
Contig_16	Contig_208	93517	237015	1	146747	Contig_208	85887	109606	23719	-
Contig_16	Contig_331	41062	96709	1	76297	Contig_331	10929	25371	14442	-
Contig_17	Contig_131	441024	635661	5660	208829	Contig_131	32148	73885	41737	-
Contig_17	Contig_155	311221	423837	1	188526	Contig_155	55102	123654	68552	+
Contig_18	Contig_109	340783	595952	612	238172	Contig_18	405247	418762	13515	-
Contig_18	Contig_114	135141	338884	1	229077	Contig_114	47500	98832	51332	+
Contig_19	Contig_289	1	76070	52290	117244	Contig_19	10877	26925	16048	-
Contig_19	Contig_319	463564	598156	1	109868	Contig_319	19698	28487	8789	-
Contig_20	Contig_298	8116	160069	1	159583	Contig_20	25148	32280	7132	+
Contig_20	Contig_298	8116	160069	1	159583	Contig_298	91658	101497	9839	+
Contig_20	Contig_81	275035	519308	1	277101	Contig_81	235467	255930	20463	+
Contig_21	Contig_255	486651	582019	1	121155	Contig_255	86349	104155	17806	+
Contig_21	Contig_270	363376	488359	6296	124912	Contig_270	12721	20846	8125	+
Contig_21	Contig_270	363376	488359	6296	124912	Contig_21	392923	409384	16461	+
Contig_23	Contig_216	375753	529872	1	141928	Contig_23	469761	494328	24567	-
Contig_25	Contig_184	166162	356263	1054	158533	Contig_25	237978	283780	45802	+
Contig_27	Contig_265	300343	404657	1	127446	Contig_265	61436	107580	46144	+
Contig_28	Contig_104	1	213903	1765	242909	Contig_104	99574	123422	23848	+
Contig_28	Contig_95	395201	472584	12615	69499	Contig_28	413621	449854	36233	+
Contig_28	Contig_443	234014	308104	1	82353	Contig_443	18119	31965	13846	+
Contig_30	Contig_220	263480	328845	23748	141506	Contig_220	89455	108697	19242	-
Contig_31	Contig_243	217523	369885	6724	131475	Contig_31	281242	313564	32322	+

Contig_32	Contig_227	1	63055	1	65597	Contig_227	18095	27037	8942	-
Contig_32	Contig_168	71810	254998	185	174718	Contig_168	69251	77928	8677	+
Contig_33	Contig_473	187911	269554	1	76059	Contig_33	205288	218038	12750	+
Contig_33	Contig_473	187911	269554	1	76059	Contig_33	230827	246221	15394	+
Contig_33	Contig_473	187911	269554	1	76059	Contig_473	42826	52711	9885	+
Contig_33	Contig_302	268544	394756	1	115335	Contig_33	287924	298009	10085	+
Contig_34	Contig_315	99264	211816	1	110835	Contig_34	115232	126910	11678	+
Contig_35	Contig_188	22049	160652	1	90946	Contig_35	106282	114886	8604	-
Contig_35	Contig_188	22049	160652	1	90946	Contig_35	55234	68474	13240	-
Contig_35	Contig_83	177502	438066	1577	275929	Contig_35	189040	218235	29195	-
Contig_37	Contig_86	69743	356984	1	268794	Contig_37	258396	303086	44690	+
Contig_37	Contig_86	69743	356984	1	268794	Contig_86	156976	189481	32505	+
Contig_38	Contig_329	57591	137070	18698	106858	Contig_329	34513	51253	16740	-
Contig_40	Contig_105	76894	298450	1	211749	Contig_105	118228	130555	12327	+
Contig_40	Contig_105	76894	298450	1	211749	Contig_40	156725	169525	12800	+
Contig_41	Contig_432	205742	282318	1	84472	Contig_432	18373	29051	10678	+
Contig_41	Contig_432	205742	282318	1	84472	Contig_41	226664	237228	10564	+
Contig_44	Contig_156	72238	214844	1	167983	Contig_156	15859	21122	5263	+
Contig_44	Contig_156	72238	214844	1	167983	Contig_156	26126	37578	11452	+
Contig_46	Contig_143	110440	326244	1	197827	Contig_143	130412	161990	31578	+
Contig_48	Contig_133	154213	344974	23065	206653	Contig_48	199045	215608	16563	-
Contig_50	Contig_304	8155	145918	1	115038	Contig_50	23975	39144	15169	-
Contig_54	Contig_321	101346	211713	1	108742	Contig_321	35224	49477	14253	+
Contig_56	Contig_136	32921	279146	38571	203372	Contig_56	79691	156719	77028	+
Contig_72	Contig_75	1	183536	89784	296310	Contig_75	194370	214660	20290	+
Contig_78	Contig_96	5052	252065	1	242082	Contig_96	102926	163896	60970	+
Contig_87	Contig_152	18953	199359	1	167861	Contig_87	146125	179835	33710	-
Contig_95	Contig_147	87698	254320	12498	193676	Contig_147	86210	93538	7328	+
Contig_93	Contig_170	14233	209017	1	170848	Contig_93	105061	125291	20230	+
Contig_89	Contig_250	1	70121	4318	131961	Contig_250	23602	57954	34352	+
Contig_89	Contig_250	1	70121	4318	131961	Contig_250	86132	125530	39398	+

Contig_100	Contig_276	96075	223132	1	123128	Contig_100	124054	141653	17599	-
Contig_100	Contig_276	96075	223132	1	123128	Contig_276	65645	71630	5985	-
Contig_117	Contig_142	1129	227761	1	186773	Contig_117	108948	163253	54305	+
Contig_129	Contig_200	1641	104553	1	149307	Contig_200	21391	59479	38088	+
Contig_121	Contig_305	62719	217554	1	114956	Contig_121	72212	117069	44857	+
Contig_148	Contig_284	37268	135772	1	121663	Contig_284	21766	44890	23124	+
Contig_158	Contig_191	1	162794	2009	155210	Contig_158	29371	37264	7893	+
Contig_197	Contig_237	7706	130305	21863	133784	Contig_197	84196	110961	26765	-
Contig_182	Contig_253	3061	144221	7	131626	Contig_253	109599	114479	4880	+
Contig_182	Contig_253	3061	144221	7	131626	Contig_182	83080	93117	10037	+
Contig_307	Contig_433	4364	107121	1	81352	Contig_307	34651	45148	10497	+
Contig_307	Contig_433	4364	107121	1	81352	Contig_307	47691	64801	17110	+
Contig_447	Contig_435	1	77321	6858	84009	Contig_435	18317	41989	23672	-
Contig_447	Contig_435	1	77321	6858	84009	Contig_435	31386	42272	10886	-

Supplementary Table 7. Enriched GO terms of tandem gene duplicates in *S. lepidophylla*.

pathway ID	pathway description	observed gene count	false discovery rate
GO.0004674	protein serine/threonine kinase activity	161	1.13E-100
GO.0004672	protein kinase activity	167	4.61E-100
GO.0001653	peptide receptor activity	97	8.08E-89
GO.0004675	transmembrane receptor protein serine/threonine kinase activity	96	3.42E-88
GO.0004888	transmembrane signaling receptor activity	97	4.84E-86
GO.0020037	heme binding	101	8.31E-85
GO.0004497	monooxygenase activity	89	2.15E-82
GO.0031625	ubiquitin protein ligase binding	97	9.93E-82
GO.0005506	iron ion binding	90	4.30E-75
GO.0016705	oxidoreductase activity	90	7.61E-66
GO.0004871	signal transducer activity	98	5.08E-63
GO.0016709	oxidoreductase activity	63	2.10E-58
GO.1901363	heterocyclic compound binding	321	1.97E-54
GO.0097159	organic cyclic compound binding	321	2.20E-54
GO.0005524	ATP binding	181	3.87E-50
GO.0035639	purine ribonucleoside triphosphate binding	190	1.85E-49
GO.0032550	purine ribonucleoside binding	190	2.56E-49
GO.0003824	catalytic activity	356	8.78E-49
GO.0032555	purine ribonucleotide binding	190	1.00E-48
GO.0032553	ribonucleotide binding	191	1.86E-48
GO.0043167	ion binding	307	1.94E-48
GO.0030246	carbohydrate binding	72	1.29E-46
GO.0016740	transferase activity	195	1.01E-42
GO.0005515	protein binding	123	2.28E-42
GO.0043168	anion binding	195	7.59E-40
GO.0000166	nucleotide binding	192	6.27E-39
GO.0036094	small molecule binding	196	8.03E-39
GO.0016491	oxidoreductase activity	105	1.24E-25
GO.0005488	binding	325	2.26E-22
GO.0005516	calmodulin binding	30	4.06E-20
GO.0046914	transition metal ion binding	92	9.42E-19
GO.0003674	molecular_function	389	6.13E-18
GO.0016713	oxidoreductase activity	8	2.55E-10
GO.0018685	alkane 1-monooxygenase activity	7	9.65E-10
GO.0015112	nitrate transmembrane transporter activity	9	3.52E-09
GO.0004366	glycerol-3-phosphate O-acyltransferase activity	6	2.14E-07
GO.0005200	structural constituent of cytoskeleton	9	6.96E-07
GO.0003682	chromatin binding	21	8.43E-07
GO.0070330	aromatase activity	5	9.73E-07

GO.0090447	glycerol-3-phosphate 2-O-acyltransferase activity	5	9.73E-07
GO.0008422	beta-glucosidase activity	12	7.02E-06
GO.0004737	pyruvate decarboxylase activity	4	3.07E-05
GO.0042936	dipeptide transporter activity	4	3.07E-05
GO.0080054	low-affinity nitrate transmembrane transporter activity	4	3.07E-05
GO.0000293	ferric-chelate reductase activity	5	4.59E-05

Supplementary Table 8. Enriched KEGG pathways of tandem gene duplicates in *S. lepidophylla*.

pathway ID	pathway description	observed gene count	false discovery rate
945	Stilbenoid	46	5.23E-54
903	Limonene and pinene degradation	45	6.66E-53
1110	Biosynthesis of secondary metabolites	85	1.34E-17
1120	Microbial metabolism in diverse environments	45	1.94E-14
940	Phenylpropanoid biosynthesis	28	1.32E-13
460	Cyanoamino acid metabolism	13	2.09E-07
73	Cutin	8	3.65E-06
966	Glucosinolate biosynthesis	7	1.21E-05
1100	Metabolic pathways	90	1.22E-05
561	Glycerolipid metabolism	8	0.00159
360	Phenylalanine metabolism	11	0.00528
4145	Phagosome	8	0.0261
564	Glycerophospholipid metabolism	8	0.0305
1210	2-Oxocarboxylic acid metabolism	7	0.0477

Supplementary Table 9. Enriched GO terms of tandem gene duplicates in *S. moellendorffii*.

pathway ID	pathway description	observed gene count	false discovery rate
GO.0020037	heme binding	133	2.27E-105
GO.0097159	organic cyclic compound binding	484	4.34E-75
GO.1901363	heterocyclic compound binding	484	4.34E-75
GO.0004497	monooxygenase activity	93	1.14E-69
GO.0005506	iron ion binding	95	6.14E-63
GO.0016705	oxidoreductase activity	95	2.15E-53
GO.0043167	ion binding	426	1.36E-49
GO.0005488	binding	537	6.26E-44
GO.0004674	protein serine/threonine kinase activity	122	3.75E-39
GO.0004672	protein kinase activity	128	1.31E-38
GO.0016491	oxidoreductase activity	145	4.42E-29
GO.0001653	peptide receptor activity	60	9.95E-29
GO.0003674	molecular_function	615	1.15E-28
GO.0004675	transmembrane receptor protein serine/threonine kinase activity	59	2.86E-28
GO.0004888	transmembrane signaling receptor activity	61	2.86E-28
GO.0046914	transition metal ion binding	142	2.86E-28
GO.0043531	ADP binding	39	3.39E-28
GO.0032559	adenyl ribonucleotide binding	192	6.47E-27
GO.0005524	ATP binding	190	7.72E-27
GO.0003700	transcription factor activity	143	1.03E-26
GO.0031625	ubiquitin protein ligase binding	60	2.73E-25
GO.0032550	purine ribonucleoside binding	197	2.68E-24
GO.0004872	receptor activity	62	7.35E-24
GO.0032555	purine ribonucleotide binding	197	8.43E-24
GO.0035639	purine ribonucleoside triphosphate binding	195	1.07E-23
GO.0032553	ribonucleotide binding	198	1.75E-23
GO.0004601	peroxidase activity	39	5.96E-20
GO.0019899	enzyme binding	61	4.63E-19
GO.0043168	anion binding	206	1.13E-17
GO.0004871	signal transducer activity	62	5.54E-17
GO.0046872	metal ion binding	221	8.63E-17
GO.0000166	nucleotide binding	201	1.27E-16
GO.0060089	molecular transducer activity	63	2.71E-16
GO.0005516	calmodulin binding	30	1.11E-14
GO.0003677	DNA binding	149	3.10E-13
GO.0003824	catalytic activity	379	1.18E-12
GO.0015238	drug transmembrane transporter activity	14	3.75E-09

GO.0005515	protein binding	90	9.61E-09
GO.0018685	alkane 1-monooxygenase activity	7	2.03E-08
GO.0050105	L-gulonolactone oxidase activity	7	2.03E-08
GO.0015293	symporter activity	23	4.52E-08
GO.0030246	carbohydrate binding	37	1.07E-07
GO.0015112	nitrate transmembrane transporter activity	9	1.61E-07
GO.0044212	transcription regulatory region DNA binding	23	2.16E-07
GO.0015291	secondary active transmembrane transporter activity	32	2.02E-06
GO.0005215	transporter activity	81	2.39E-06
GO.0070330	aromatase activity	5	7.85E-06
GO.0016740	transferase activity	153	3.44E-05
GO.0022804	active transmembrane transporter activity	40	3.44E-05
GO.0003676	nucleic acid binding	155	5.56E-05
GO.0005355	glucose transmembrane transporter activity	9	7.54E-05

Supplementary Table 10. Enriched KEGG pathways of tandem gene duplicates in *S. moellendorffii.*

pathway ID	pathway description	observed gene count	false discovery rate
945	Stilbenoid	49	3.76E-50
903	Limonene and pinene degradation	48	3.17E-49
940	Phenylpropanoid biosynthesis	53	5.06E-31
360	Phenylalanine metabolism	36	1.42E-19
1110	Biosynthesis of secondary metabolites	116	2.18E-19
1120	Microbial metabolism in diverse environments	48	2.15E-09
591	Linoleic acid metabolism	6	1.46E-05
460	Cyanoamino acid metabolism	13	2.95E-05
73	Cutin	8	8.72E-05

Supplementary Table 11. Summary of Orthogroup classifications.

	**Arabidopsis**	**Rice**	**Oropetium**	**Poplar**	***S. lepodiophylla***	***S. moellendorffiii***	**Grape**
Number of genes	27,416	39,049	28,437	42,950	27,204	22,285	26,346
Number of genes in orthogroups	22,530	24,890	19,245	32,069	17,134	16,466	19,532
Number of unassigned genes	4,886	14,159	9,192	10,881	10,070	5,819	68,14
Percentage of genes in orthogroups	82.2	63.7	67.7	74.7	63	73.9	74.1
Percentage of unassigned genes	17.8	36.3	32.3	25.3	37	26.1	25.9
Number of orthogroups containing species	10,352	11,523	11,356	11,087	9,090	8,976	10,242
Percentage of orthogroups containing species	68.2	76	74.9	73.1	59.9	59.2	67.5
Number of species-specific orthogroups	37	37	37	38	70	62	10
Number of genes in species-specific orthogroups	432	235	112	364	841	434	43
Percentage of genes in species-specific orthogroups	1.6	0.6	0.4	0.8	3.1	1.9	0.2

Supplementary Table 12. Enriched GO terms in orthogroups with overrepresentation in Angiosperms.

pathway ID	pathway description	observed gene count	false discovery rate
GO.0043531	ADP binding	65	9.85E-64
GO.0015299	solute:proton antiporter activity	28	8.71E-20
GO.0005451	monovalent cation:proton antiporter activity	22	1.04E-19
GO.0015077	monovalent inorganic cation transmembrane transporter activity	50	5.62E-19
GO.0015078	hydrogen ion transmembrane transporter activity	39	1.74E-17
GO.0010333	terpene synthase activity	21	2.67E-17
GO.0022890	inorganic cation transmembrane transporter activity	58	3.30E-16
GO.0008553	hydrogen-exporting ATPase activity	11	2.94E-12
GO.0030552	cAMP binding	14	9.85E-12
GO.0030553	cGMP binding	14	9.85E-12
GO.0080043	quercetin 3-O-glucosyltransferase activity	22	6.38E-11
GO.0080044	quercetin 7-O-glucosyltransferase activity	22	6.38E-11
GO.0030247	polysaccharide binding	18	8.46E-11
GO.0005261	cation channel activity	20	4.11E-08
GO.0032559	adenyl ribonucleotide binding	157	5.68E-08
GO.0022836	gated channel activity	20	1.88E-07
GO.0005249	voltage-gated potassium channel activity	12	9.43E-07
GO.0047893	flavonol 3-O-glucosyltransferase activity	8	1.10E-06
GO.0030145	manganese ion binding	14	2.39E-06
GO.0005179	hormone activity	13	2.41E-06
GO.0000287	magnesium ion binding	24	2.60E-06
GO.0004674	protein serine/threonine kinase activity	68	7.55E-06
GO.0045735	nutrient reservoir activity	14	7.55E-06
GO.0015276	ligand-gated ion channel activity	10	1.64E-05
GO.0080031	methyl salicylate esterase activity	5	3.08E-05
GO.0015075	ion transmembrane transporter activity	52	4.64E-05
GO.0005524	ATP binding	141	6.26E-05

Supplementary Table 13. Enriched KEGG pathways in orthogroups with overrepresentation in Angiosperms.

pathway ID	pathway description	observed gene count	false discovery rate
960	alkaloid biosynthesis	12	1.60E-05
4120	Ubiquitin mediated proteolysis	19	0.00625
4626	Plant-pathogen interaction	20	0.029

Supplementary Table 14. Enriched GO terms in orthogroups with overrepresentation in *Selaginella*.

pathway ID	pathway description	observed gene count	false discovery rate
GO.0048207	vesicle targeting, rough ER to cis-Golgi	2	0.00071
GO.0048208	COPII vesicle coating	2	0.00071
GO.0090114	COPII-coated vesicle budding	2	0.00071
GO.0051650	establishment of vesicle localization	2	0.00506
GO.0042744	hydrogen peroxide catabolic process	3	0.025

Supplementary Table 15. Enriched KEGG pathways in orthogroups with overrepresentation in *Selaginella*.

pathway ID	pathway description	observed gene count	false discovery rate
360	Phenylalanine metabolism	3	0.0154
940	Phenylpropanoid biosynthesis	3	0.0185

Supplementary Table 16. Tandem gene duplicates induced during desiccation.

Gene ID	# in tandem array	Annotation	RNAseq (TPM)					
			Sel_0 hr	Sel_1hr	Sel_6hr	Sel_24hr	Sel_120hr	Sel_De24hr
Sle_012709	2	dehydrin	19860.3	25245.9	26737.73	8707.127	9400.985	34810.97
Sle_012707	2	dehydrin	13236.13	15757.27	16746.37	6955.453	4596.25	18232.6
Sle_002382	3	ELIP	5947.803	5237.77	5421.657	794.7973	103.3718	2305.329
Sle_014211	8	ELIP	5352.087	5143.617	6006.04	752.3097	178.3909	2185.167
Sle_021620	3	(R,S)-reticuline 7-O-methyltransferase	5315.37	3689.2	3473.317	1085.689	1849.709	2206.557
Sle_015016	3	ELIP	4898.673	5739.09	5425.667	740.7287	105.3366	945.0467
Sle_000102	2	ribulose bisphosphate carboxylase small chain 1B	3605.3	2626.357	3213.947	2660.827	8606.74	3876.18
Sle_014207	8	ELIP	3569.097	4366.277	3440.22	426.0397	118.6547	1227.424
Sle_021626	3	actinoporin	3559.582	2380.933	2279.9	2265.313	1068.367	1427.953
Sle_014212	8	ELIP	2441.887	2533.25	2492.473	374.6957	140.3598	1361.857
Sle_015015	3	ELIP	2251.303	2068.557	2262.563	420.9243	68.6149	617.4533
Sle_003590	2	caleosin	2147.48	2589.75	2804.44	730.4543	366.5695	1959.687
Sle_012388	2	oleosin	2112.603	2798.82	2596.277	951.7137	552.873	3184.917
Sle_007889	2	oleosin	2025.057	2005.73	1972.26	277.3123	124.0364	862.3717
Sle_003538	2	hypothetical	1581.513	1943.3	2097.227	1344.647	1133.284	1549.75
Sle_010581	2	Oxygen-evolving enhancer protein 1	1488.293	1260.893	1111.571	1803.993	3057.67	899.311
Sle_009642	2	hedgehog protein	1454.837	2607.613	2396.331	1331.431	2615.815	1949.023
Sle_014210	8	ELIP	1422.937	1821.233	1724.272	183.1617	43.08995	136.7193
Sle_014209	8	ELIP	1404.191	1702.453	1395.273	180.9858	31.13525	272.7183
Sle_024166	2	HSP	1135.277	908.5877	1192.659	834.1273	41.78465	107.8245
Sle_005885	2	quinone oxidoreductase	996.786	992.4773	892.972	585.2337	461.4305	806.7607
Sle_000989	2	glutathione S transferase	974.811	911.782	909.6337	792.3367	846.0455	943.241
Sle_002390	2	oxidoreductase	926.5847	967.0173	888.632	190.2527	273.411	618.333

Sle_014206	8	40S ribosome	900.94	963.3943	818.2637	1307.9	546.836	1243.437
Sle_007803	2	HSP	857.2963	771.9047	835.075	723.8253	244.2675	385.7747
Sle_010272	2	sucrose transport protein SUT2	817.2857	833.618	914.1527	252.4047	315.6265	731.058
Sle_014203	8	elip	807.556	1291.413	792.083	279.8271	54.1481	205.5453
Sle_004563	2	stress responsive protein [Zea mays]	765.2367	610.4017	612.0637	375.0683	410.62	648.98
Sle_008375	2	ricin B-like lectin	641.525	727.319	760.3923	315.8133	356.314	825.8673
Sle_016272	7	probable flavin-containing monooxygenase 1	612.8717	2262.248	9594.367	9276.867	14300.55	428.471
Sle_002978	2	hypothetical protein	611.5091	276.9507	191.0872	60.68521	443.166	190.816
Sle_008542	2	HSP	557.379	461.984	697.5757	344.969	10.74791	46.91903
Sle_018502	2	HSP	551.5403	377.5227	512.9093	952.5367	458.298	313.9707
Sle_005883	2	carbonic anhydrase	548.5533	444.768	692.5117	443.5267	1185.645	817.666
Sle_016705	4	dehydration-responsive element binding protein 2	540.7077	420.4193	565.7143	417.1933	183.3795	396.4697
Sle_018503	2	Thiosulfate sulfurtransferase 16	488.6333	534.1037	421.8857	470.275	425.729	525.384
Sle_007925	3	ATP-binding protein	477.592	333.8573	352.7967	410.229	424.3175	642.7573
Sle_004564	2	ricin B-like lectin	469.3307	345.292	263.5537	239.3123	372.57	254.5963
Sle_011308	2	60S ribosomal proteins	439.5363	467.8273	445.3357	670.7167	393.49	527.5097
Sle_006185	3	SUMO-conjugating enzyme SCE1	413.3453	374.9207	348.166	310.4457	304.7875	346.5297
Sle_011169	4	Cold-regulated plasma membrane	393.2983	406.875	478.29	191.6063	499.5685	851.328
Sle_010829	3	unknown	381.436	186.5667	156.2967	272.765	412.1925	606.4583
Sle_025239	2	S-adenosyl homocysteine hydrolase	380.8653	333.4577	350.9593	1118.381	1524.445	1100.355
Sle_021623	3	actinoporin	380.129	440.6813	430.626	334.4513	393.3145	251.3643
Sle_002346	2	putative nitrite transporter	375.6333	288.591	290.0983	150.1027	431.65	400.3697
Sle_010053	4	short-chain dehydrogenase TIC 32	367.848	290.8877	306.983	279.592	193.0535	328.652
Sle_015196	3	outer membrane protein porin	337.2352	264.1202	595.4063	2092.997	9.013955	686.4511
Sle_024087	7	Aldo-keto reductase family 4	335.28	315.7547	346.5427	163.385	259.194	507.7413
Sle_005398	2	unknown	327.121	365.18	408.4043	143.244	36.91195	181.976
Sle_010252	2	unknown	323.442	349.2993	337.4917	347.4463	143.0615	237.3693
Sle_006321	2	isoflavone reductase	323.2913	291.1133	328.4953	1025.229	658.172	657.5103
Sle_007185	3	Ras-related protein RABC1	312.3637	335.0633	320.2547	200.957	125.3675	288.7383

Sle_009916	2	Cytochrome c oxidase copper chaperone 1	307.7633	382.6257	363.619	937.8653	235.7095	495.1983
Sle_017181	4	steroleosin	304.212	340.173	422.2007	122.164	91.70155	172.712
Sle_009295	2	ribosomal protein S10	297.231	374.112	315.1053	239.423	287.6905	226.3807
Sle_006214	2	glutamate decarboxylase	288.8383	246.322	253.3633	196.6037	294.1725	183.073
Sle_011721	2	tyrosine kinase family protein	287.3563	275.9683	274.6417	372.2803	418.0155	284.404
Sle_000221	4	unknown	280.86	257.857	280.8367	281.1837	261.741	511.865
Sle_009022	2	unknown	265.6893	160.635	349.7583	196.0207	261.304	179.709
Sle_011162	2	small auxin up RNA1	263.647	282.6707	267.7683	346.1753	157.263	340.4007
Sle_020291	2	Beta glucosidase 40	249.52	207.6163	184.364	127.1325	415.047	381.8013
Sle_014760	2	beta-glucosidase 12	242.7647	224.772	262.5537	201.3837	676.7105	327.655
Sle_005285	2	Heat shock protein 81	242.1087	133.1723	311.77	905.796	384.854	424.5317
Sle_004977	2	40S ribosomal protein	235.5503	249.4237	247.5023	360.935	190.98	318.9813
Sle_006197	2	phosphoglycerate kinase	232.5847	217.1067	215.4597	143.712	274.415	292.2877
Sle_002366	4	cytokinin hydroxylase	231.8587	189.6013	201.673	171.1223	332.079	221.4533
Sle_026138	4	unknown	213.0207	316.5467	316.574	192.7877	138.5315	112.1706
Sle_007765	2	sterol 8,7-isomerase	207.8693	226.2993	204.8843	99.31023	117.681	144.1957
Sle_004193	2	Select seq ref|XP_020528179.1|	207.0383	193.7113	222.5623	1222.7	269.0735	336.3983
Sle_007747	2	unknown	200.613	156.717	183.557	103.4894	45	192.3417
Sle_003575	2	CAAX amino terminal protease	199.2977	167.0157	121.1442	178.3223	145.6205	261.9347
Sle_002964	2	Zinc finger, FYVE-type	197.5907	132.418	141.9527	61.1329	118.524	56.10377
Sle_007672	2	caffeoyl-CoA O-methyltransferase	185.32	204.07	220.7437	299.687	1046.636	277.148
Sle_001415	8	protein TAB2 homolog	180.1617	160.2293	135.983	243.136	286.376	129.7263
Sle_003998	2	glutaredoxin	178.5453	162.476	158.218	156.041	54.02505	143.439
Sle_006364	2	chalcone-flavonone isomerase	172.7947	176.3793	129.7297	492.2077	485.7365	796.425
Sle_000103	3	Alpha-crystallin domain 32.1, putative isoform 2	168.0667	178.7177	171.8193	78.7705	33.7112	129.5683
Sle_013083	5	protein FEZ-like	166.9843	165.5843	152.7297	207.98	393.666	116.9981
Sle_007899	2	ELIP	166.341	154.6141	104.1234	41.13103	23.3967	49.76063
Sle_001411	2	Alpha/beta-Hydrolases	164.2207	151.205	130.3163	136.255	90.87535	168.8063
Sle_003925	2	Universal stress protein	161.541	140.7293	144.437	173.2333	74.89375	152.8547

Sle_012971	2	PITH domain-containing protein 1	151.741	157.795	165.894	114.055	87.9862	133.4263
Sle_003678	2	LEA1	148.0903	190.1143	315.8	150.8417	29.7065	94.00193
Sle_025334	2	sucrose transport protein SUC3	143.078	117.6204	129.7793	43.34137	57.8068	77.41013
Sle_014364	3	ZIP family metal transporter	142.358	144.078	156.6483	68.0439	57.68425	117.4427
Sle_001250	2	short-chain dehydrogenase	119.594	136.3597	106.364	69.78187	70.44895	68.4713
Sle_005027	4	ubiquitin-protein ligase	119.3033	106.3108	115.5925	86.44987	78.97225	114.122
Sle_021282	4	peroxisomal adenine nucleotide carrier 1	112.5925	113.7027	107.483	61.2	47.42825	77.6918
Sle_007594	2	EPF-type Cis2-His2 zinc finger transcription factor	112.4168	92.47633	81.0742	52.33887	37.26695	54.06327
Sle_011054	5	UV radiation resistance protein/autophagy-related protein 14	104.628	93.51503	91.67127	43.83487	46.3518	72.66547
Sle_003782	2	Exportin-1	104.3811	112.2093	104.4566	66.7854	113.262	74.07007

Supplementary Table 17. Summary of LEA proteins in *Selaginella* genomes.

LEA subfamily	*S. lepidophylla*	*S. moellendorffii*
LEA1	3	1
LEA2	31	19
LEA4	2	2
LEA5	14	8
Dehydrin	4	2
SMP	11	4
**Total**	**65**	**36**

Supplementary Table 18. Relative water content of *S. lepidophylla* during the rehydration time-course.

Timepoint (post rehydration)	Relative water content (% ± SD)
0 hr	11.2 ± 1.1
1 hr	14.3 ± 4.9
2 hr	21.4 ± 2.7
6 hr	28.5 ± 3.4
12 hr	43.2 ± 3.2
24 hr	68.4 ± 6.5
120 hr	75.3 ± 2.3
24 hr (dehydrated)	15.3 ± 2.5
